# Tracing Corrosive Damage in Human Teeth: A Forensic Pilot Study of Household Agents Using Stereomicroscopy, SEM-EDX and Ground Sections

**DOI:** 10.3390/molecules31111797

**Published:** 2026-05-23

**Authors:** Larisa Adela Udriştioiu, Marius Enăchescu, Alexia Ecaterina Cârstea, George Cristian Curcă, Mihaela-Monica Popa, Mihai Andrei

**Affiliations:** 1 Department of Legal Medicine and Bioethics, Faculty of Medicine, “Carol Davila” University of Medicine and Pharmacy, 050474 Bucharest, Romania; larisa.udristioiu@umfcd.ro (L.A.U.);; 2 National Institute of Legal Medicine “Mina Minovici”, 042122 Bucharest, Romania; 3 Center for Surface Science and Nanotechnology, University Politehnica of Bucharest, 060042 Bucharest, Romania; 4 Academy of Romanian Scientists, StreetIlfov 3, 030167 Bucharest, Romania; 5 Faculty of Dentistry, “Carol Davila” University of Medicine and Pharmacy, Dionisie Lupu Street, No. 37, District 2, 020021 Bucharest, Romania; 6 Department of Embryology and Microbiology, Faculty of Dentistry, “Carol Davila” University of Medicine and Pharmacy, 050474 Bucharest, Romania

**Keywords:** forensic traces, body concealment, corrosive substances, SEM, EDX, ground sections, stereomicroscopy, dental and hard tissues

## Abstract

Teeth may retain forensic value after chemical exposure, yet the effects of commercially available corrosive agents remain insufficiently characterized. This study evaluated short-term alteration patterns in human teeth exposed to household acidic and alkaline products available on the Romanian market. Five extracted mandibular third molars were analyzed, including four experimental teeth and one control. Each experimental tooth was fully immersed for 48 h in a different agent: hydrochloric acid descaler, sodium hypochlorite bleach, mixed hydrochloric/sulfuric acid descaler, or sodium hydroxide. Morphometric changes, mass, and pH were monitored serially, while stereomicroscopy, SEM-EDX, and hard tissue ground sections were used for structural and compositional assessment. Acid-exposed teeth showed the greatest damage, with major mass loss in the hydrochloric acid and mixed-acid samples, enamel loss, and marked microstructural disruption. The mixed-acid specimen exhibited the most severe collapse and near-complete calcium/phosphorus depletion. Sodium hypochlorite produced mainly superficial and root-level alterations with relative preservation of gross morphology, whereas sodium hydroxide caused minimal dimensional change and a calcium-rich adherent surface deposit. These findings show that household corrosives produce distinct, forensically recognizable dental alteration patterns within 48 h and support an integrated pattern-recognition approach in suspected chemical concealment scenarios.

## 1. Introduction

The use of caustic or corrosive substances for criminal purposes, particularly for the concealment or destruction of human remains, is not a novel phenomenon. Although relatively uncommon, this method is considered one of the most severe forms of body concealment, being classified as grade 3 in Schneikert’s classification, alongside bricking up or embedding in concrete, dismemberment, feeding to animals, and burning [1,2]. In another classification, such acts fall under defensive mutilation, reflecting the perpetrator’s attempt to hinder recognition, identification, or reconstruction of the circumstances of death [3].

The deliberate use of chemical agents to dispose of human remains has been depicted in both academic discussions [3] and has also entered popular culture through novels and television series [4]. However, only a limited number of real case reports are documented in the forensic literature, and detailed descriptions of how these cases were managed remain scarce [3,5,6,7,8,9]. Instead, most available studies are experimental and aim to evaluate the consequences of exposure to various acids and alkalis on biological tissues [2,10,11].

Among the tissues investigated in this context, teeth occupy a particular position because they are among the most resistant structures of the human body. Their highly mineralized composition, together with the structural organization of enamel, dentine, cementum, and the root complex, makes them valuable substrates in forensic identification and postmortem analysis. Nevertheless, dental hard tissues are not immune to chemical destruction. Exposure to strong acids or alkalis may induce macroscopic, microscopic, and compositional alterations that can affect both their morphology and their forensic interpretability.

Experimental studies on teeth exposed to corrosive substances have produced heterogeneous results. This variability is related to several factors, including the type and concentration of the chemical agent, the duration of exposure, the immersion protocol, the degree of tooth maturation, and the analytical methods applied. Reported outcomes range from complete dissolution, with the shortest time reported in the literature being 8 h for hydrochloric acid [12,13,14] to more limited or superficial alterations [15,16,17]. Although sulfuric acid has been associated with body disposal by criminal organizations [18], experimental studies suggest that hydrochloric acid may cause more extensive damage to dental tissues [2,12,15,16,17,19,20,21].

Initial observations in many studies were based primarily on the macroscopic examination of teeth following exposure to corrosive agents. More recent investigations have incorporated advanced analytical techniques, such as scanning electron microscopy (SEM) [16,21] and energy-dispersive spectroscopy (EDX) [21], to characterize microstructural changes. For example, SEM investigations have demonstrated a lamination-like effect on dental tissues after prolonged exposure (approximately 66 h) to 37% hydrochloric acid in maxillary molars [21]. The same authors also reported a faster penetration of acid into immature molars compared with fully developed teeth [21]. Beyond structural alteration, previous studies have also explored whether chemically exposed teeth may retain forensic value, including attempts to recover DNA after exposure to hydrochloric acid, nitric acid, and sodium hydroxide [15,20,22]. More recently, artificial intelligence-based approaches have been investigated, particularly for identification through the three-dimensional reconstruction of dental patterns [23].

Despite these contributions, the available literature remains heterogeneous, and relatively few studies have integrated macroscopic, microscopic, elemental, and hard-tissue section-based observations within the same experimental framework. Moreover, a broader analysis of cases involving corrosive substances indicates that with few exceptions [23], perpetrators in real-life situations tend to use readily available commercial products rather than specialized industrial chemicals [4]. This aspect is particularly relevant from a forensic perspective, as household products may contain complex mixtures of active substances and additives, producing alteration patterns that differ from those observed with laboratory-grade reagents.

In this context, the present forensic pilot study aimed to evaluate the effects of several commercially available household corrosive agents from the Romanian market on human dental hard tissues. The study combined serial pH assessment, mass and morphometric monitoring, stereomicroscopy, SEM-EDX analysis, and hard-tissue ground sections in order to characterize the macroscopic, microscopic, and compositional changes induced by these agents. This work is part of a broader series of forensic experimental investigations addressing the impact of chemical agents on biological structures [24].

## 2. Results

### 2.1. pH Measurements of Experimental Solutions

Table 1 shows the pH measurements of the solutions used in the experiment at the baseline (0 h) and at the end of the experiment, after 48 h. For the first and third solutions, the initial pH value of 0 confirmed their strongly acidic nature. After 48 h, the pH of both solutions increased slightly, likely reflecting tooth demineralization and the release of mineral ions into the surrounding medium. The greatest variation was observed in the second solution, indicating a marked shift from a strongly alkaline environment toward a near-neutral pH. In contrast, the fourth solution was strongly alkaline at the baseline and remained relatively stable throughout the experiment, with only minimal variation after 48 h.

### 2.2. Morphometric Analysis and Mass Measurements

#### 2.2.1. Mass Measurements

The change in mass differed among the experimental teeth, S1–S4, at the end of the 48-h immersion in corrosive solutions (Table 2). Apart from tooth S4, which recorded a 2.94% increase in mass, and tooth S0, whose mass remained constant, the rest of the teeth experienced a decrease in mass of 44.79% for S1 and 52.45% for tooth S3, respectively. The steepest mass loss was recorded for teeth S1 and S3, which were immersed in corrosive acid solutions.

A further change in mass was recorded when the teeth were left to dehydrate at room temperature for 48 h prior to SEM analysis. For tooth S1, a mass of 0.61 g was recorded, indicating a 42.45% decrease; for S2, a mass of 1.7 g, with a 9.57% loss; for S3, a mass of 0.58 g, with a 40.21% loss; and for the last tooth, S4, a mass of 1.86 g, showing a mass reduction of 17.96%.

#### 2.2.2. Morphometric Analysis

##### Mesiodistal and Buccolingual Diameters

S1 and S3 both showed progressive diameter reduction. In S1, the mesiodistal diameter decreased from 11.6 mm to 9.22 mm, and 6 h after immersion, the value no longer showed any significant change, while the buccolingual dimension fell from 10.11 mm to 7.8 mm and reached a plateau phase even earlier, at 3 h. S3 followed the same pattern with mesiodistal values stabilizing at 8.7 mm and buccolingual at 8.15 mm, and the changes remained insignificant between 12 and 18 h (Table 2). The plateau in both specimens marks the point of complete enamel loss at the anatomical equator. Once the dentin is exposed, no further diameter reduction occurs, as dentin demonstrates greater resistance to the erosive agents. S2 and S4 showed no diameter change at any time point, indicating the absence of detectable equatorial enamel loss throughout the observation period (Table 2).

##### Crown Length and Total Tooth Length

S1 crown length decreased from 8.17 mm to 7.40 mm, and remained roughly the same at 12 h; total length decreased from 17.91 mm to 17.43 mm over the same interval. In S3, crown length showed a steeper reduction from 7.93 mm to 6.67 mm, reaching its minimum at 18 h, while the total length reached a stable value earlier, at approximately 12 h. The earlier stabilization of total length points to a non-uniform distribution of enamel thickness along the long axis of the tooth. S2 and S4 remained unchanged across both parameters (Table 2).

### 2.3. Stereomicroscopy Evaluation

**Figure 1:**
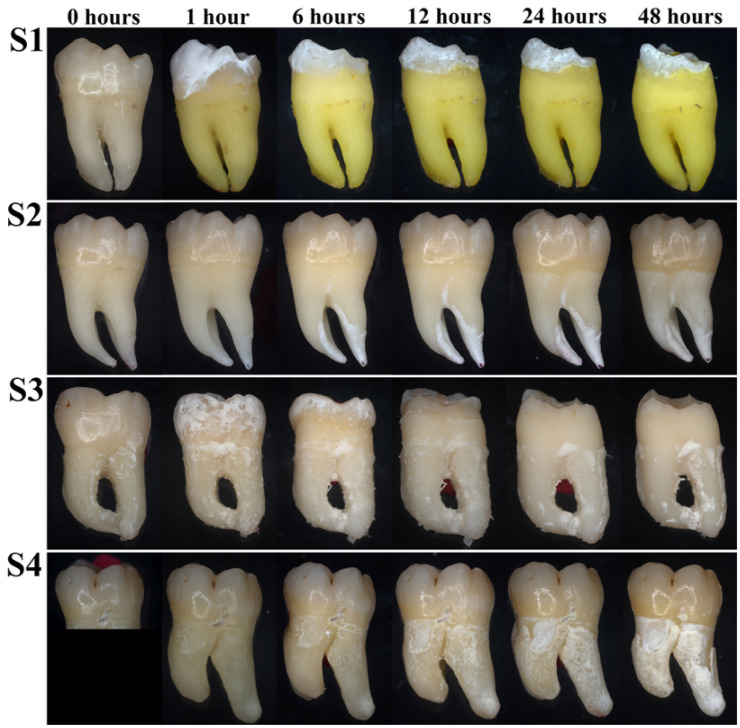
The figure shows stereomicroscope images of teeth S1–S4 taken at various time points during the experiment: 0 h, 1 h, 6 h, 12 h, 24 h, and 48 h. Due to an error, the complete photograph of tooth S1 from the buccal view is missing, so an image of the crown was added.

The teeth used in the experiment (S1–S4) exhibited, macroscopically at 0 h, a typical crown and root morphology for both enamel and cementum (Figure 1, S1–S4, 0 h). The enamel had a shiny and smooth appearance, with the presence of the perikymata, while the cementum had a dull, pale yellow appearance.

In the case of tooth S1, acid attack at the crown level was significant, and after only one hour of immersion in the solution, the enamel was removed at the enamel–cement junction. In Figure 1, the buccal view revealed enamel removal at the distobuccal cusp, and striae of Retzius were visible near the exposed dentin. The enamel had a matte, chalky-white appearance and carious lesions were still visible. Areas of demineralization were more pronounced at the distal cusps and on the lingual surface. At the root level, the cementum had an intense yellow color, due to the demineralized solution, which exhibited this color. Six hours after immersion, the enamel had been removed in the two-thirds closest to the cementoenamel junction. The matte white appearance, with a loss of surface detail, persisted, and areas of demineralization were visible. Between 12 and 48 h, no significant changes were observed, as demineralization was slowed by the solution becoming saturated with minerals removed from the tooth.

In the case of tooth S2, the enamel appeared normal at 0 h (Figure 1). One hour after immersion, the enamel exhibited a more pronounced gloss, while at the cementum level, a much duller appearance was observed apically, and the apex was widened. Between 6 and 48 h, no loss was observed at the crown level, with the enamel remaining glossy; at the root level, the demineralization surface was present primarily in the apical third and in the area of the root furcation. The apical root areas tended to widen, but the process was slow.

The most striking changes could be observed in tooth S3. The acidic solution acted rapidly within 1 h, which could be seen on the crown surface, with demineralization defects present across the entire enamel surface. After approximately 24 h, the enamel had completely disappeared, except for a small area located on the occlusal surface of the mesio-lingual cusp. After 24 h, the glossy appearance of the coronal dentin was noticeable. Regarding the root, its matte appearance changed after 24 h, and deposits could be observed on the cementum (Figure 1, S3).

For tooth S4, due to an error, the full image at 0 h was missing and replaced with an image of the crown. Changes in the enamel were minimal, including on surfaces where carious lesions were present. The enamel became much glossier as time passed, and perikymata could be observed on it throughout the whole period. In contrast, a compact layer could be observed on the roots, particularly in the apical region. The accumulation of this layer caused the mass of tooth S4 to be greater at the end of the experiment compared to the rest of the teeth (Figure 1, S4, 48 h).

### 2.4. SEM and EDX Analysis

#### 2.4.1. Tooth S0

**Figure 2:**
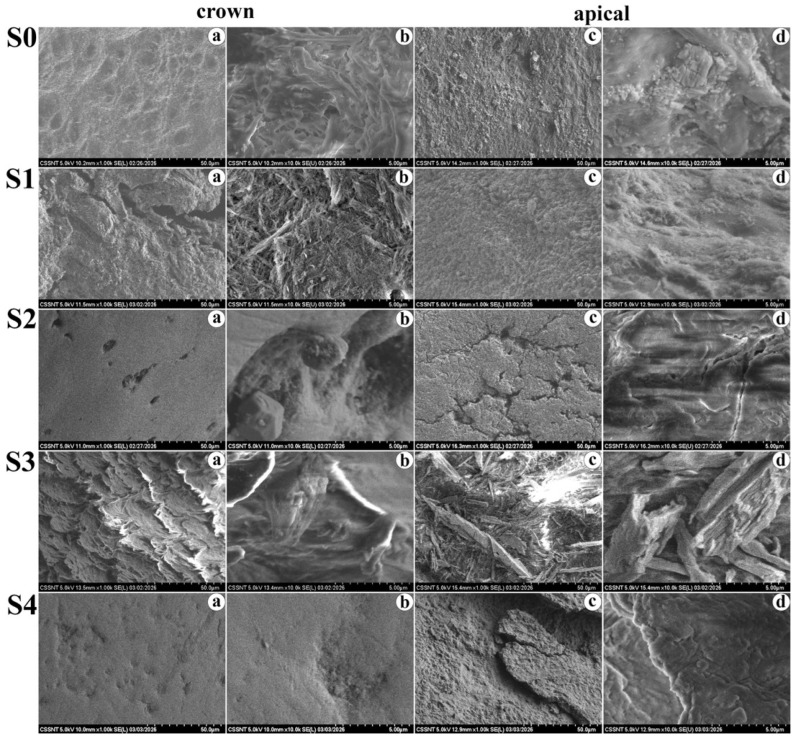
SEM micrographs of teeth S0–S4 showing the crown and apical regions. For each region, panels (**a**,**c**) represent the images obtained at ×1k magnification, while panels (**b**,**d**) show higher-magnification (×10k) views of the same area.

**Figure 3:**
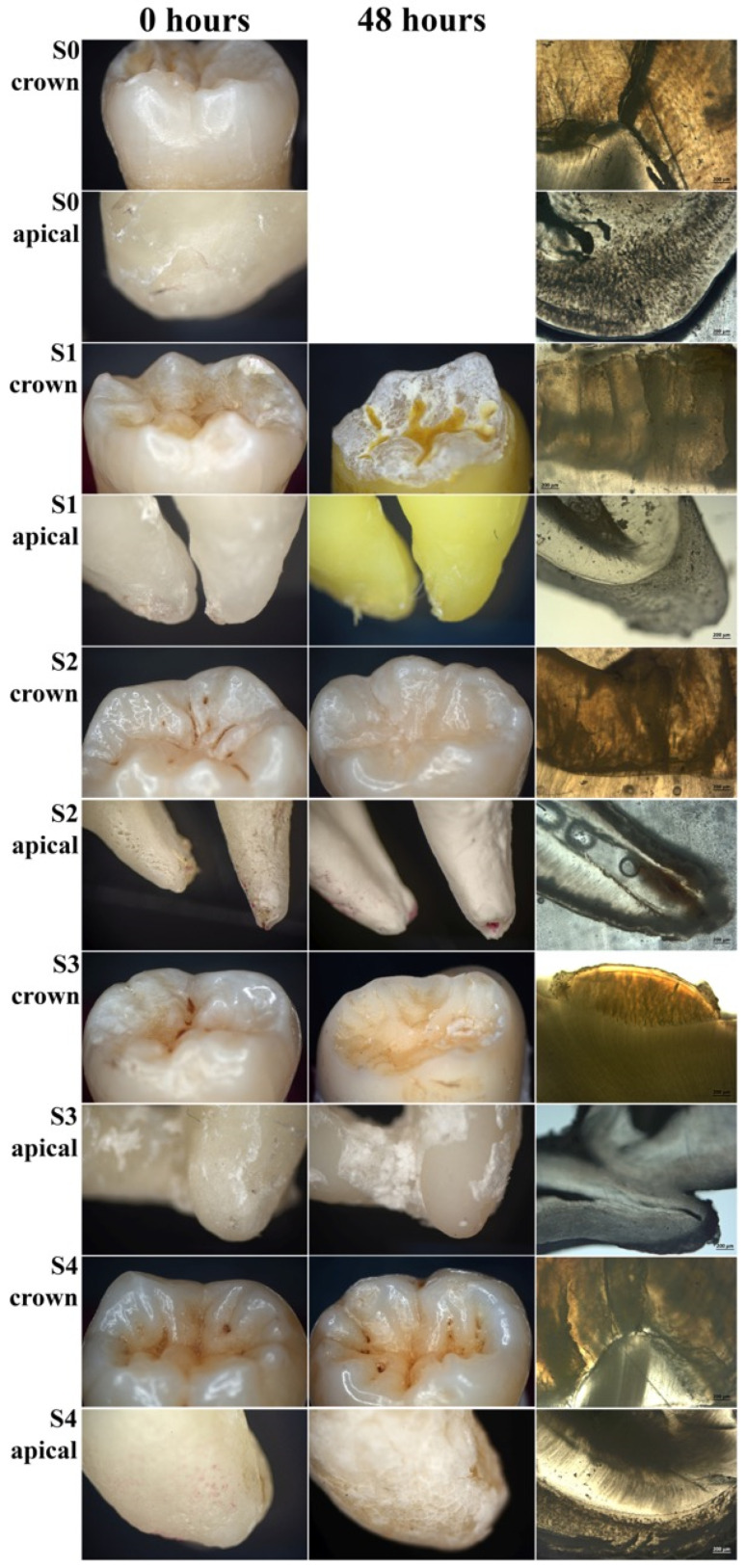
Stereomicroscopic and light microscopic images of ground sections for teeth S1–S4 and S0.

Crown SEM micrographs revealed a relatively compact and homogeneous surface without evidence of etching, delamination, or overt structural disruption (Figure 2; S0, a and b). EDX of the coronal surface was dominated by carbon, oxygen, and nitrogen, with only limited and variable detection of calcium and phosphorus (Table 3). A similar pattern was observed on the root surface, where SEM showed a relatively uniform fibrillar to finely textured microtopography. In the apical region, however, the surface became more heterogeneous, with irregular granular and discontinuous deposits (Figure 3; S0, c and d). EDX from this region remained predominantly organic-rich but showed more variable calcium and phosphorus values, together with focal sulfur and chlorine signals (Table 3).

#### 2.4.2. Tooth S1

SEM examination of the residual enamel revealed marked surface alteration, including irregular topography, microcavities, microfissures, and loss of prismatic architecture. At higher magnification, an acicular microstructure was observed (Figure 2; S1, b). EDX of the residual enamel remained mineral-dominant, with calcium and phosphorus well-represented and chlorine detectable in several spectra (Table 3). Coronal dentin showed more severe structural disruption, with irregular surface morphology, microcracking, loss of tubular architecture, and dense acicular to bundle-like formations at higher magnification, consistent with exposed or partially reorganized hydroxyapatite crystals following acid-induced demineralization (Figure 2; S1, a). EDX of this region likewise remained calcium- and phosphorus-rich, but with reduced carbon and nitrogen, consistent with a partially preserved hydroxyapatite framework. In contrast, the root was substantially more altered (Table 3). Root areas showed increasingly disorganized, amorphous, and irregular surfaces, with loss of recognizable dentinal architecture (Figure 2; S1, c and d). In these regions, EDX was dominated by carbon, nitrogen, and oxygen, whereas calcium and phosphorus were markedly reduced and heterogeneously distributed; chlorine was focally detected in advanced areas (Table 3).

#### 2.4.3. Tooth S2

SEM micrographs showed a non-uniform pattern of alteration. On the crown, some fields showed only mild superficial micropits (Figure 2; S2, a and b). On the root, the changes were more diffusely distributed and consisted mainly of microcracking, lamellar fragmentation, increased roughness, and superficial disorganization of the outer layer (Figure 2; S2, c and d). EDX indicated that the mineral phase was largely preserved, with calcium and phosphorus peaks and low but persistent sodium signals detected. Only one focal coronal area showed a markedly heterogeneous chemical profile, with focal chlorine detected, a relative reduction in calcium and phosphorus, and increased carbon-oxygen contribution, compatible with localized mineral loss and/or superimposed residual surface material (Table 3).

#### 2.4.4. Tooth S3

SEM demonstrated extensive collapse and disorganization of both crown and root surfaces, with replacement of the normal compact architecture by lamellar, folded, porous, fibrillar, and filamentous residual structures. Coronal areas showed severe disruption of the enamel/dentin surface, with near-complete loss of the normal topography and abundant adherent altered material (Figure 2; S3 a and b). EDX spectra in these areas revealed near-total depletion of calcium and phosphorus, while carbon and nitrogen predominated; focal chlorine was also detected (Table 3). Apical root areas showed a similarly severe pattern, with diffuse corrosive alteration and replacement of the superficial substrate by residual non-mineral material. The apical areas were also profoundly altered (Figure 2; S3 a and b). SEM showed a severely degraded substrate overlain by abundant plate-like and needle-like deposits and severe surface disorganization with collapsed fibrillo-lamellar material and irregular adherent deposits; EDX spectra revealed near-complete loss of phosphate, only trace-to-focal calcium detection, and increased sulfur (Table 3).

#### 2.4.5. Tooth S4

On the crown, SEM revealed heterogeneous superficial alteration characterized by diffuse micropitting, granular irregularity, and loss of the typical smooth enamel appearance. No well-defined prismatic or interprismatic pattern was identified (Figure 2; S4, a and b). EDX confirmed persistence of the mineral phase, although localized reductions in calcium and relative increases in carbon, oxygen, and sodium were observed at higher magnification, indicating focal rather than uniform mineral alteration (Table 3). The root surface showed a more distinctive pattern. SEM demonstrated the presence of an adherent lamellar to plate-like surface layer/“fish-scale pattern” with cracking, structural consolidation, and partial exfoliation (Figure 2; S4, c and d). In the mid-root areas, this layer appeared more continuous and compact, whereas in the apical region, it was thinner, more fragmented, and partially detached, with transitional zones exposing the underlying substrate. EDX spectra showed reduced phosphorus, increased oxygen, persistent sodium, and an elevated Ca/P ratio, consistent with a calcium-rich secondary deposited phase (Table 3).

### 2.5. Ground Sections and Light Microscopy Evaluation

Compared to the usual ground sections, those obtained in this experimental study were sectioned from partially demineralized/altered teeth (S1–S4), with the exception of S0. The ground sections exhibited greater elasticity than those from non-demineralized teeth.

The experimental teeth, S1–S4, and the control tooth, S0, were sectioned after SEM analysis. Figure 3 shows macroscopic images (at 0 and 48 h) and microscopic images of dental ground sections for teeth S1–S4 and S0. Stereomicroscopic images were taken at 0 and 48 h, at the occlusal surfaces (from lingual view—the cusps shown are the buccal ones) and apical region (from buccal), with the exception of S0 who was only investigated macroscopic at 0 h.

S0 exhibited typical morphology at the coronal level. Perikymata, which represent the external manifestation of striae of Retzius on the outer surface of the enamel, are visible on the buccal cusps. Vertical fissures are noticeable, which may be due to dehydration of the tooth, a step that was necessary for SEM analysis. At the apical level (Figure 3, S0 apical), the apex of one of the two roots appeared normal, characterized by a certain roughness and a pale yellow color.

The ground sections of S0 revealed enamel with a normal, yellowish appearance. Striae of Retzius can be identified on the mesio-buccal cusp of the crown, which is shown in Figure 3. A prominent lamella extends across the entire thickness of the enamel to the tip of the cusp. The coronal dentin is characterized by the presence of dentinal tubules. At the apical level, the layer of cellular cementum is thick, and lacunae can be observed within its structure.

For S1, stereomicroscopic images at 0 h showed normal coronal morphology and appearance of the enamel. At 48 h, enamel was present strictly at the occlusal surface, and in many regions of it, the underlying dentin was visible due to its reduced thickness. The area of the fossae and grooves, where carious processes were present, was also demineralized (Figure 3, macroscopic images at 0 and 48 h). The microscopic image of the enamel revealed the presence of numerous lamellae extending across its entire thickness. The enamel texture was irregular, and striae of Retzius were not visible. The enamel dentin junction had an opaque appearance, and the dentinal tubules and odontoblastic processes were reduced in number. In the upper side of the image, an air bubble can be seen entrapped in the resin in which the tooth was embedded for sectioning. In the case of the root of tooth S1, a change in the structure of the cellular cementum could be observed, with the lacunae becoming blurred. The odontoblastic processes adjacent to the cemento-enamel junction were reduced, and the root dentin had a much more transparent appearance.

In the case of tooth S2, a normal crown morphology could be observed at 0 h, with carious lesions present in the grooves and fossae. In contrast, at 48 h, the carious lesions were much less pronounced following the action of the sodium hypochlorite-based solution (Figure 3, images at 0 h and 48 h). No differences were observed between 0 and 48 h following the action of the corrosive solution, as the morphology of the hard coronal tissues remained almost unchanged. In contrast, in the apical region of the two roots, a widening of the apexes was noted, as well as a much more matte and opaque color of the cementum (Figure 3, the light microscopy image). The widening of the apexes could also be observed in the light microscopy image, where the penetration of the resin into the root canal was evident. The odontoblastic processes adjacent to the root canal were reduced in number, and the dentin had a much more transparent appearance. No lacunae were observed in the cellular cementum structure.

Tooth S3 exhibited the most pronounced changes at the coronal level, with the enamel almost completely lost at the coronal level after 48 h, except for a very small area, which could be seen in the light macroscopy image. At the end of the immersion period, exposed dentin was visible at the occlusal level, and buccal, lingual, and marginal dentin ridges were observed. At the apical level, the cementum surface was much smoother at 48 h, and deposits were observed at the root level (Figure 3, macroscopic images for the crown and root, at 0 and 48 h). The island of remaining enamel was visible in the light microscopy image. The enamel was dark yellow in color, and its structure revealed numerous enamel tufts adjacent to the amelodentinal junction and lamellae with an ascending trajectory. The dentin was traversed by dentinal tubules containing odontoblastic processes. At the apical level, cementum was visible under the microscope; externally, the secondary cementum was affected by demineralization, and the lacunae in its structure were no longer identifiable.

Macroscopically, at the coronary level of S4, there were no noticeable differences between 0 and 48 h, although a slightly more pronounced luster was observed at the end of the immersion period. At the root tip, the cement appeared normal at 0 h, and at 48 h, and the root was covered by a deposit from the caustic soda solution (Figure 3, crown and apical region at 0 and 48 h). The light microscopy image of the crown of S4 revealed an enamel interspersed with numerous lamellae that ran upward from the amelodentinal junction. In the root of tooth S4, the presence of cellular cementum was observed in the light microscopic image of the hard tissue section. Histology appeared normal, which may be due to the deposit that formed at the root level and acted as a protective layer. The cellular cementum was replete with lacunae, and at the cement dentin junction, the hyaline layer of Hopewell–Smith could be observed (Figure 3, light microscopy of S4, crown and apical region).

To facilitate practical interpretation, the main alteration patterns observed for each tested agent were synthetically summarized in Table 4, integrating mass change, macroscopic findings, SEM-EDX features, ground-section observations, and the corresponding forensic implications.

## 3. Discussion

Given the extent of this phenomenon, as well as the growing public and forensic awareness of body concealment through chemical destruction—both in media representations and in organized criminal practices—and considering today’s commercial availability of products capable of producing such effects [24], an experimental study focusing on one of the most resistant human tissues, namely teeth, was warranted. The commercial products selected for this study contained some of the best-known substances with significant destructive potential for both soft and hard tissues, including hydrochloric acid, sulfuric acid [25], and from the alkaline spectrum, caustic soda. Markedly, the deliberate choice was made to investigate commercially available household products rather than professional or laboratory-grade reagents, precisely because such substances are more likely to be encountered in real-life concealment attempts.

Overall, the observed patterns could only be partially aligned with previously reported findings. However, the available literature remains markedly heterogeneous, with substantial variation in the type and concentration of the solutions used, exposure time, immersion protocol (complete or partial immersion, with or without repeated re-immersion), and overall experimental design [4].

In addition, no truly comparable studies were identified that specifically assessed the utility of ground sections for this purpose, indicating that this approach remains insufficiently explored in the current literature [26]. Such variability highlights the need for greater standardization in this field, as also noted by Bracewell and Jones [27].

Regarding the mechanism of action, acid-containing agents primarily induce dissolution of the apatite phase [28], whereas alkaline substances act mainly through hydrolytic, oxidative, or proteolytic disruption of the organic component [29,30,31]. Accordingly, a pattern-based interpretation integrating macroscopic, SEM, EDX, and ground section findings is likely to be more useful in forensic practice than reliance on just one feature.

Under stereomicroscopic examination, external changes were identified on both the crown and root surfaces, including alterations in color, transparency, and enamel surface features such as perikymata, which represent the surface manifestation of striae of Retzius [32]. Perikymata are particularly present on newly erupted teeth, giving the enamel a rougher texture, and over time, the processes of dental wear lead to their fading. In teeth S1–S4 and S0, perikymata were identified at 0 h on the buccal surfaces [33]. Only teeth S2 and S4, whose enamels were minimally affected, retained these visible features on their external surfaces throughout the experiment.

The control tooth (S0) provided a realistic biological baseline rather than an idealized mineral reference. Its preserved morphology, lack of etching or delamination, and heterogeneous carbon-, nitrogen-, and oxygen-rich spectra are consistent with an intact hydrated outer surface, particularly in the crown region, where intrinsic structural complexity and superficial organic deposits are more likely to be encountered. This observation is important from a practical perspective, because variable or even reduced surface Ca/P detection on an untreated tooth should not automatically be interpreted as evidence of chemical degradation. Comparative SEM/EDX studies have shown that normal enamel, dentine, and cementum are intrinsically heterogeneous and that external root surfaces may display a more variable elemental profile than enamel-dominated crown surfaces [25].

The HCl-exposed tooth (S1) exhibited the clearest strong-acid demineralization profile, with progressive mineral loss and more advanced disruption in dentine and root areas than in the crown. The persistence of a residual enamel with microcracks, contrasted with the marked collapse of root dentine, is consistent with the known action of hydrochloric acid, which rapidly dissolves calcium-phosphate apatite; once mineral support is removed, dentine becomes especially vulnerable because of its lower mineral content and greater organic fraction compared with enamel. We observed that the carious lesions showed relative resistance to acid attack, consistent with the findings of Aoba and Yagi [34]. However, our observations were only partially in agreement with previous reports. Unlike some published studies, none of our specimens developed a soft, spongy, or “jelly-like” consistency after exposure [2]. Moreover, within the investigated timeframe, dissolution did not progress to complete structural destruction or to the point at which the tooth or hard tissue would become unrecognizable [11,25]. Exposure of the pulp chamber and the presence of visible residues in the container were also not observed [21]. Finally, we did not identify the “laminated” appearance repeatedly described after hydrochloric acid exposure in both mature and immature molars [16,21]. In forensic terms, the combination of pronounced macroscopic alteration, severe SEM disorganization, and EDX depletion of Ca and P strongly supports the interpretation as mineral-acid injury, while also indicating that altered but still recognizable dental substrate may persist during the first 48 h of exposure.

The NaOCl-exposed tooth (S2), in contrast, followed a different pathway. Here, the overall tooth outline remained largely preserved, while the observed changes were predominantly superficial and heterogeneous, including focal crown injury and more diffuse microcracking, roughening, and lamellar fragmentation at the root level, without extensive global mineral loss. This pattern accords with the established action of sodium hypochlorite as a strong oxidizing and proteolytic/deproteinizing agent, directed mainly toward the organic matrix, particularly dentinal collagen [35,36,37,38]. Its effect on carious lesions was also noted [39]. Experimental studies have shown that NaOCl may alter the dentinal mineral content and Ca/P ratio to some extent; however, its dominant effect remains matrix degradation and ultrastructural disorganization. From a medico-legal standpoint, this distinction is relevant because a tooth may remain macroscopically recognizable despite genuine chemical exposure, and the combined pattern of preserved morphology, focal SEM injury, and limited mineral alteration may support exposure to hypochlorite-containing agents.

The commercial mixed-acid product containing hydrochloric and sulfuric acid displayed the most aggressive profile in the present series. Macroscopically, the tooth S3 rapidly lost its normal appearance; SEM demonstrated severe surface collapse and disorganization; and EDX revealed near-complete depletion of the calcium-phosphate phase in multiple areas, leaving predominantly carbon-, nitrogen-, and oxygen-rich residual material. This pattern is best interpreted as mixed-acid demineralization, likely driven primarily by the hydrochloric acid component, with additional chemical complexity introduced by sulfuric acid. The sulfur-containing signals may be reasonably discussed as compatible with exposure to the sulfuric component or with sulfur-containing secondary surface residues; however, EDX alone cannot establish the precise identity of any such phase. From a medico-legal perspective, this finding is particularly relevant because it shows that even when normal dental morphology is rapidly obliterated, residual analyzable material may still persist at 48 h, preserving potential evidential value.

The caustic soda tooth (S4) differed clearly from the acid-exposed samples. Its defining pattern was not classical acid etching with diffuse Ca/P depletion, but rather caustic alkaline surface alteration, characterized by diffuse micropitting, granular irregularity, fissuring, and apparent surface reorganization or deposition. This interpretation is further supported by the fact that S4 was the only tooth reported to show a slight increase in weight, suggesting the retention of exogenous material and/or surface deposition rather than straightforward tissue loss alone, broadly consistent with previous observations reported in the literature [2,40]. Strong alkalis act mainly through the hydrolytic degradation of organic components and caustic disruption of tissue architecture, which helps explain why substantial exposure may occur without the dramatic demineralization pattern seen with strong acids. In practical forensic terms, a grossly preserved tooth showing fissuring, surface deposits, and altered sodium-rich superficial chemistry may therefore be more suggestive of strong alkali exposure than of acid corrosion [2].

The pH shifts recorded after 48 h of contact with dental tissue suggested that the tooth did not behave as an inert substrate, but rather as a chemically interactive system capable of modulating the surrounding medium. The most pronounced effect was observed in Solution 2 (PROMAX Active Chlorine Bleach), whose pH decreased from 11.56 to 7.59, becoming nearly neutral despite its initially strong alkalinity. This substantial shift was likely driven by the oxidative degradation of organic components, hydroxyl ion consumption in secondary reactions, atmospheric CO_2_ absorption, and phosphate-mediated buffering following hydroxyapatite dissolution, all contributing to displacement of the OCl^−^/HOCl equilibrium toward less alkaline conditions. In comparison, the acidic solutions exhibited only minor pH increases, while the caustic soda solution remained largely stable. These findings indicate that the buffering capacity of dental tissues is solution-dependent and particularly evident in sodium hypochlorite-containing media.

Across all chemically exposed teeth, the Ca/P ratio requires cautious interpretation. In this context, Ca/P is best discussed using atomic percentages, since the reference value for hydroxyapatite is an atomic/molar ratio rather than a weight ratio. Even so, Ca/P should not be treated as a standalone diagnostic marker. In altered specimens, the absolute reduction in calcium and phosphorus, together with SEM morphology and the broader elemental profile, is often more informative than the ratio alone, particularly when superficial deposits, residual organic material, or exogenous elements may distort the spectrum obtained from the outermost analyzed surface [41].

Ground sections are an effective way to examine hard dental tissues under a microscope, as teeth can be sectioned without prior processing, and the enamel, dentin, and cementum remain intact. Compared to tooth processing methods that involve demineralization in acidic solutions, the technique of ground sections remains somewhat unstandardized at present. A novelty in the experimental study was the sectioning of partially demineralized dental hard tissues. Elements of the structure of these tissues were identified and characterized. Post-immersion changes in the corrosive solutions were assessed at 48 h, with the exception of S0, the control tooth, using dental hard tissue sections. In the case of acidic solutions, changes in the enamel of teeth S1 and S3 were evident, as evidenced by the loss of detail and the disappearance of Retzius striae—brown bands that reflect the deposition of enamel in the form of incremental lines during amelogenesis [33]. At the root level, in teeth S1, S2, and S3, the acellular cementum exhibited a loss of lacunae containing entrapped cementocytes [42]. Other changes involved reducing the number of dentinal tubules adjacent to the amelodentinal and cementodentinal junctions. The ground sections confirmed the clear evidence of changes in dental hard tissue induced by acid-based solutions, as demonstrated in teeth S1 and S3, supporting the findings of the stereomicroscopic and SEM analyses.

Taken together, the present findings support a practically useful forensic pattern-recognition framework. HCl was associated with severe acid demineralization and progressive structural collapse, particularly in dentine and root tissue. The HCl/H_2_SO_4_ commercial product generated the most destructive mixed-acid pattern, with profound mineral loss and chemically complex residues. NaOCl largely preserved global tooth morphology while producing focal alkaline-oxidative/proteolytic damage, whereas NaOH was more suggestive of preserved gross form with fissuring and surface reorganization rather than frank acid-type dissolution. Such distinctions may prove useful in future casework when recovered teeth are visibly altered but not completely destroyed and the examiner seeks to narrow the likely chemical environment involved.

Beyond their value for pattern recognition, these findings also have implications for the residual identificatory potential of chemically altered teeth. Severe chemical modification of enamel and coronal dentine does not necessarily imply a complete loss of forensic utility, particularly when root structures remain at least partially preserved. This is relevant because teeth are not only resistant morphological substrates, but are also potential sources of genetic material for human identification. Corte-Real et al. showed that the tooth is topographically heterogeneous in terms of DNA recovery, with the apical root portion providing the highest DNA yield and representing a preferential region for the preparation of mineralized dental tissue for molecular analysis and forensic identification [43]. Accordingly, in cases of suspected chemical body concealment, the assessment of altered teeth should include not only the description of visible chemical damage, but also careful evaluation of preserved root segments, especially the apical region, as these may retain residual potential for DNA analysis even when the crown is severely compromised.

Several limitations should nevertheless be acknowledged. Most importantly, this was a pilot study with a very small sample size, with only one tooth assigned to each exposure condition and one control specimen; therefore, no replication was available for the individual chemical agents, precluding an assessment of within-group variability and limiting the inferential strength of the findings. Consequently, this design does not allow for robust comparative conclusions regarding product-specific effects. In addition, individual teeth may differ substantially in their pre-existing biological and environmental characteristics, including degree of mineralization, previous fluoride exposure, enamel thickness, carious history, occlusal or mechanical wear, and other structural or pathological changes, all of which may influence resistance to chemical dissolution or surface alteration. Consequently, the observed alteration patterns should be interpreted as exploratory and descriptive rather than as definitive substance-specific signatures.

All specimens were clinically extracted mandibular third molars, which limits the generalizability of the results to other tooth types, different degrees of maturation, restored teeth, and teeth with pre-existing wear, carious lesions, periodontal changes, or other alterations commonly encountered in forensic cases.

Further limitations relate to the experimental model itself. The specimens were fully immersed outside the jaw and without surrounding soft tissues. Although this limitation was already inherent to the experimental design, it is important to stress that in real-life forensic situations, tooth roots would usually be covered or at least partially protected by gingival tissues, periodontal structures, and alveolar bone. These surrounding structures may influence the degree of direct contact between corrosive agents and the root surface, the rate and distribution of chemical alteration, and the interaction between the solution and dental tissues. Therefore, the present model may overestimate direct root exposure compared with some forensic scenarios. In addition, the teeth were repeatedly removed, rinsed, photographed, and re-immersed. Moreover, SEM/EDX analysis was performed on intact outer surfaces rather than on sectioned samples. Although these conditions allowed for the controlled observation of surface alterations, future refinements would further enhance the forensic applicability of the model and allow for a more robust assessment of deeper structural changes and reaction products. Future studies should therefore include larger series, experimental designs involving continuous immersion, periodic solution renewal, and teeth originating from different taphonomic settings, as well as section-based SEM/EDX, micro-CT, complementary histology [44], and structural analytical methods capable of characterizing secondary deposits more precisely.

## 4. Materials and Methods

### 4.1. Study Design and Dental Samples

The present experimental in vitro descriptive-comparative pilot study investigated the morphological and compositional alterations occurring in human dental tissues following exposure to commonly available acidic and alkaline household substances.

A total of five extracted human mandibular third molars were included in the analysis. Four teeth were used as experimental samples and were designated S1, S2, S3, and S4, while one tooth served as the negative control specimen (S0). All experimental samples corresponded to mandibular third molars with the tooth numbering 3.8 (The Fédération Dentaire Internationale (FDI) World Dental Numbering System [45]), and the control sample corresponded to tooth 4.8. Only teeth with preserved coronal and radicular structures were included in the study. Localized carious lesions restricted to fossae and grooves were present, with no associated structural destruction or appreciable loss of hard dental tissue. Consequently, limited areas of demineralization related to cariogenic activity were present in some specimens, reproducing a substrate closer to real-life conditions. After extraction, each tooth was maintained in 10% neutral buffered formalin [46].

### 4.2. Corrosive Solutions and Experimental Exposure

The experimental teeth were individually immersed in commercially available household products containing either acidic or alkaline active substances. Each tooth was placed in a separate container containing 10 mL of the experimental solution, ensuring complete immersion of the dental structure. The total experimental duration was 48 h.

Four different solutions were used in the experiment. The first solution consisted of Misavan Descaler (Misavan Trading, Iași, Romania), a commercially available descaling agent containing hydrochloric acid in a concentration of 5–15%, as well as fragrance and preservatives. The second solution was PROMAX Active Chlorine Bleach (PROMAX-ACB) (Romchim, Tg. Mureș, Romania), a bleaching product containing sodium hypochlorite in concentrations ranging between 1–5%, together with stabilizing agents. The third solution consisted of PROMAX Floral Descaler (PROMAX-FL) (Romchim, Tg. Mureș, Romania), a descaling product containing hydrochloric acid in concentrations of 15–30% and sulfuric acid in concentrations of 1–5%, in addition to non-ionic surfactants present in concentrations below 1% and fragrance compounds. The fourth solution consisted of caustic soda flakes (Kynita, Vâlcea, Romania) dissolved in water at a 1:1 mass-to-volume ratio (1 g/mL) (Table 5).

Each solution was assigned to one experimental tooth, with the purpose of observing the specific effects produced by different corrosive agents. The control specimen was not exposed to any chemical solution and was preserved for comparative morphological and compositional analyses (Table 5). During the experiment, to determine the mass and length, the teeth were removed from the demineralizing solutions, rinsed with water, and dried under a gentle stream of air. The pH of the demineralizing solutions was measured initially, before the teeth were immersed in the solutions, and at the end of the experiment, after 48 h. The pH was measured using a C1020 multi-parameter analyzer (Consort, Turnhout, Belgium).

### 4.3. Morphometric Analysis

Baseline measurements were performed prior to immersion (t = 0 h), followed by measurements at 1, 3, 6, 12, 18, 24, 36, and 48 h. Measurements of the buccolingual and mesiodistal diameters were taken at the anatomical equator, which is an imaginary line connecting the points of maximum convexity on the lateral surfaces of the tooth [47]. The measurement of the crown height was taken from the tip of the mesiobuccal cusp to the cementoenamel junction, and the measurement of the total length was taken at the level of the mesiobuccal cusp to the tip of the mesial root. All measurements were performed using a precision caliper S_Cal EVO Basic (Sylvac, Crissier, Switzerland).

These morphometric parameters were monitored simultaneously by two investigators during the experimental period in order to identify dimensional alterations associated with progressive dental demineralization induced by the corrosive solutions.

### 4.4. Mass Measurements

In order to evaluate the quantitative loss of mineralized dental tissue, the mass of each experimental tooth was measured throughout the duration of the experiment. The measurements were performed using an EWJ precision laboratory balance (Kern&Sohn, Balingen, Germany).

Each experimental tooth (S1–S4) was weighed at predefined intervals throughout the experimental period. The measurements were recorded at the following time points: 0 h, 1 h, 3 h, 6 h, 12 h, 18 h, 24 h, 36 h, and 48 h. This sequential weighing protocol allowed for the monitoring of progressive reductions in mass associated with the dissolution of mineralized dental components under the action of the experimental solutions. The mass of the experimental teeth was also determined after they had been dehydrated for SEM analysis.

### 4.5. Stereomicroscopy Evaluation

Morphological alterations occurring on the external surfaces of the teeth were documented using stereomicroscopic examination. Observations were performed at the same experimental intervals used for the mass measurements, namely at 0, 1, 3, 6, 12, 18, 24, 36, and 48 h.

Stereomicroscopy allowed for the visualization of progressive structural changes affecting both the enamel and root surfaces during exposure to the demineralizing agents. Images were obtained from multiple orientations in order to document the progressive degradation of the dental structures and the morphological modifications occurring over time. A Stemi 2000-C stereomicroscope (Zeiss, Oberkochen, Germany) was used in conjunction with a KL 1500 LCD external light source (Zeiss, Oberkochen, Germany). The images were captured using a digital single reflex camera (Nikon, Tokyo, Japan).

### 4.6. SEM and EDX Analysis

Following the completion of the experimental exposure period, the external morphology of the dental tissues was investigated using scanning electron microscopy (SEM). In addition to morphological examination, elemental composition was evaluated using energy dispersive X-ray spectroscopy performed with an EDX analytical system. This analysis enabled the identification of changes in the mineral composition of the dental surfaces associated with the demineralization process. The results obtained from the experimental teeth were compared with that obtained from the control specimen in order to assess the extent of mineral loss and compositional alteration. A Hitachi SU 8230 scanning electron microscope (Hitachi High-Tech Corporation, Tokyo, Japan) equipped with an EDX Oxford detector-analyzer (Oxford Instruments, High Wycombe, UK) was used to perform sample analysis. After the 48 h immersion, the teeth were dried gradually over a 48-h period at room temperature to avoid accelerated dehydration, which could lead to artifacts resulting from volumetric shrinkage, fracturing, and cracking [48].

### 4.7. Ground Sections

After completion of the experimental procedures, all teeth were sectioned in order to obtain hard tissue slices suitable for light microscopy examination. These sections allowed for the investigation of structural changes occurring within the internal layers of dental tissues. Sectioned teeth were partially demineralized, and compared to conventional ground sections, they exhibited greater elasticity.

The resulting sections were examined using light microscopy, enabling the assessment of alterations affecting enamel, dentin, and cementum architecture following exposure to corrosive chemical agents. This complementary analysis provided additional information regarding the depth and pattern of demineralization induced by the experimental solutions. To prepare ground sections, the teeth were embedded in EpoFix resin (Struers, Ballerup, Denmark). The embedded teeth were then mounted in using a SMART CUT 6010 cutting machine (UKAM, Valencia, CA, USA). The cutting speed was 1000 revolutions per minute (rpm), and the load was approximately 400–500 g. A #4BC1 diamond disc (UKAM, Valencia, CA, USA) was used for sectioning, and the specimens were cooled using Cooli Additive Plus coolant (Struers, Ballerup, Denmark).

### 4.8. Light Microscopy Evaluation

The ground sections were examined using an Axiostar Plus light microscope (Zeiss, Oberkochen, Germany) and a 5x A-Plan objective (Zeiss, Oberkochen, Germany). Images were captured using an Axiocam Color 105 camera (Zeiss, Oberkochen, Germany) and the software used was Zen Blue (Zeiss, Oberkochen, Germany).

## 5. Conclusions

The present study demonstrates that even within a relatively short 48-h exposure interval, commercially available corrosive and caustic agents can produce distinct and forensically recognizable patterns of dental alteration. Rather than leading uniformly to complete destruction, the tested substances generated agent-specific profiles, ranging from marked acid-driven demineralization and structural collapse in the HCl- and mixed-acid-exposed teeth to predominantly superficial oxidative/proteolytic and caustic surface changes in the NaOCl- and NaOH-exposed specimens, with the preservation of gross morphology to varying degrees. To the best of our knowledge, the simultaneous application of stereomicroscopy, SEM-EDX, and ground dental section analysis in the forensic investigation of household corrosive-exposed teeth has not been previously reported in the literature. At the same time, the results highlight the need for caution in interpretation, particularly with regard to Ca/P ratios and superficially altered elemental profiles as well as the need for greater methodological standardization across studies.

At the same time, acid-based solutions caused the most significant loss of tooth mass and the most noticeable changes in the hard dental tissues. Despite its limitations, this study shows that chemically altered teeth may retain diagnostically useful morphological and compositional signatures during the early stages of exposure, thereby providing potentially relevant evidential clues for the reconstruction of suspected chemical concealment scenarios.

## Figures and Tables

**Table 1:** The pH of the solutions was measured at the beginning and end of the experimental period.

Solution	pH at 0 h	pH at 48 h
1st solution (Misavan Descaler)	0	0.36
2nd solution (PROMAX-ACB)	11.56	7.59
3rd solution (PROMAX-FL)	0	0.81
4th solution (Caustic soda and water)	13.7	13.51

**Table 2:** The changes in width, buccolingual diameter, mesiodistal diameter, total tooth height, and crown height are presented for the 48-h experimental period at 0, 1, 3, 6, 12, 18, 24, 36, and 48 h.

Tooth	Measurements	0 h	1 h	3 h	6 h	12 h	18 h	24 h	36 h	48 h
S1	Mass (g)	1.92	1.6	1.37	1.27	1.2	1.13	1.18	1.06	1.06
	Mesiodistal diameter (mm)	11.6	10.69	9.85	9.33	9.24	9.22	9.22	9.22	9.22
	Buccolingual diameter (mm)	10.11	10	7.8	7.8	7.8	7.8	7.8	7.8	7.8
	Tooth length (mm)	17.91	17.73	17.67	17.5	17.44	17.44	17.44	17.43	17.43
	Crown length (mm)	8.17	8.04	7.83	7.5	7.42	7.42	7.42	7.41	7.40
S2	Mass (g)	1.88	1.88	1.88	1.88	1.88	1.88	1.88	1.88	1.88
	Mesiodistal diameter (mm)	11.7	11.7	11.7	11.7	11.7	11.7	11.7	11.7	11.7
	Buccolingual diameter (mm)	9.5	9.5	9.5	9.5	9.5	9.5	9.5	9.5	9.5
	Tooth length	19.19	19.88	19.81	19.78	19.75	19.72	19.7	19.69	19.69
	Crown length	7.99	7.99	7.99	7.99	7.99	7.99	7.99	7.99	7.99
S3	Mass (g)	2.04	1.95	1.8	1.62	1.38	1.13	1.08	0.98	0.97
	Mesiodistal diameter (mm)	11.32	11.28	10.85	10.3	8.97	8.7	8.7	8.7	8.7
	Buccolingual diameter (mm)	9.58	9.54	9.05	8.2	8.15	8.15	8.15	8.15	8.15
	Tooth length	18.59	18.56	18.5	18.31	18.30	18.30	18.28	18.28	18.28
	Crown length	7.93	7.24	7.16	6.79	6.67	6.67	6.67	6.67	6.67
S4	Mass (g)	2.38	2.38	2.36	2.36	2.38	2.40	2.41	2.43	2.45
	Mesiodistal diameter (mm)	11.58	11.58	11.58	11.58	11.58	11.58	11.58	11.58	11.58
	Buccolingual diameter (mm)	10.25	10.25	10.24	10.24	10.25	10.25	10.25	10.25	10.25
	Tooth length	16.4	16.4	16.4	16.4	16.4	16.4	16.42	16.44	16.45
	Crown length	7.55	7.55	7.55	7.55	7.55	7.55	7.55	7.55	7.55

**Table 3:** EDX atomic percentages for each tooth in the crown (C) and apical (A) regions at ×1k and ×10k magnifications (Mgf).

Tooth	Region	Mgf	C	N	O	Na	P	Ca	Cl	S	Ag	Mg
**S0**	C	1	62.05	8.88	27.65	0.56	0.38	0.38	-	-	-	-
		10	73.33	10.97	14.46	0.48	0.59	0.17	-	-	-	-
	A	1	69.92	6.72	20.58	0.42	0.26	2.10	-	-	-	-
		10	70.07	2.86	22.62	0.25	1.55	2.65	-	-	-	-
**S1**	C	1	11.36	1.59	50.52	0.55	12.88	23.10	-	-	-	-
		10	11.56	3.31	52.70	1.04	10.33	20.06	1.00	-	-	-
	A	1	62.97	9.55	22.84	0.84	0.95	2.13	0.73	-	-	-
		10	50.48	9.87	28.20	0.33	3.36	7.76	-	-	-	-
**S2**	C	1	38.86	-	60.41	-	0.73	-	-	-	-	-
		10	44.89	-	47.25	-	0.71	7.16	-	-	-	-
	A	1	19.35	-	49.79	0.72	11.35	17.81	-	-	-	0.98
		10	16.20	3.49	47.42	0.63	11.80	19.42	-	-	-	1.04
**S3**	C	1	56.01	20.26	22.86	0.22	0.10	0.28	-	-	0.27	-
		10	68.10	15.26	14.68	0.52	0.23	0.52	0.70	-	-	-
	A	1	35.27	12.11	37.64	0.06	0.43	5.61	-	8.29	0.60	-
		10	38.48	6.48	26.46	0.06	0.76	14.51	-	13.25	-	-
**S4**	C	1	19.65	-	34.80	1.03	15.14	29.37	-	-	-	-
		10	18.73	4.11	46.98	0.31	18.62	11.25	-	-	-	-
	A	1	15.63	1.94	48.00	0.87	8.65	17.57	-	-	-	-
		10	11.84	1.34	46.67	0.73	12.44	26.99	-	-	-	-

**Table 4:** The results of the experimental analyses are summarized below.

Sample/Agent	Mass Change After 48 h	Macroscopic/Stereomicroscopic Pattern	SEM-EDX Pattern	Ground Sections Findings	Main Forensic Implication
**S0—** **Control**	No mass change	Preserved crown and root morphology; normal enamel and cementum appearance	Preserved microstructure; baseline heterogeneous elemental profile without chemical injury pattern	Normal hard dental tissue morphology, with identifiable enamel, dentine, cementum, Retzius striae, dentinal tubules, and cementocyte lacunae	Reference pattern for comparison; highlights that normal dental tissues may show intrinsic structural and elemental heterogeneity
**S1—Hydrochloric acid descaler/HCl 5–15%**	−44.79%	Extensive enamel loss, especially near the cementoenamel junction; exposed dentine; matte/chalky-white enamel; pronounced crown and root alteration	Marked acid-induced structural disruption; residual enamel microcracks; coronal dentine disorganization; root surface collapse; heterogeneous Ca/P depletion, especially at root level	Altered crown and root morphology; irregular enamel, loss of Retzius striae, reduced dentinal tubules/odontoblastic processes, blurred cementum lacunae	Pattern compatible with strong mineral-acid injury; altered but still recognizable dental substrate may persist after 48 h
**S2—Sodium hypochlorite bleach/NaOCl 1–5%**	0% during immersion	Gross crown morphology preserved; enamel remained glossy; root changes mainly apical, with widening and matte/opaque cementum	Predominantly superficial and heterogeneous alteration; mild crown micropitting; root microcracking, roughening, and lamellar fragmentation; mineral phase largely preserved with limited compositional disturbance	Minimal coronal hard-tissue alteration; apical root changes, widening of apexes, reduced odontoblastic processes, more transparent dentine, absence of cementum lacunae	Chemical exposure may be present despite preserved gross morphology; pattern suggests oxidative/proteolytic action rather than frank acid demineralization
**S3—Mixed acid descaler/HCl 15–30% + H_2_SO_4_ 1–5%**	−52.45%	Most severe macroscopic alteration; rapid loss of normal appearance; almost complete enamel loss; exposed dentine; marked loss of substance; root deposits	Severe surface collapse and disorganization; lamellar/folded/porous residual structures; near-complete Ca/P depletion in coronal areas; sulfur-containing deposits/signals in apical regions	Most pronounced coronal changes; residual enamel island; altered enamel with tufts and lamellae; externally demineralized secondary cementum; loss of identifiable lacunae	Most destructive pattern in the series; supports mixed-acid corrosive exposure; residual analyzable material may persist despite severe morphological obliteration
**S4—Sodium hydroxide/NaOH**	+2.94%	Limited gross loss; crown largely preserved with increased luster; root surface covered by compact deposit, especially apically	Heterogeneous crown alteration with micropitting and granular irregularity; adherent lamellar/plate-like root layer; persistent sodium; elevated Ca/P pattern compatible with calcium-rich secondary deposition	Limited crown alteration; relatively preserved root histology; cellular cementum with lacunae; deposit may have acted as a protective layer	Pattern more suggestive of strong alkali exposure than acid corrosion; preserved gross morphology with surface deposition may still carry diagnostic information

**Table 5:** Commercial name and chemical composition of the experimentally used solutions.

	Commercial Name	Chemical Composition	Immersed Tooth
1	Misavan Descaler	Hydrochloric Acid 5–15% Fragrance Preservative	S1
2	PROMAX Active Chlorine Bleach (PROMAX-ACB)	Sodium Hypochlorite 1–5% Stabilizers	S2
3	PROMAX Floral Descaler (PROMAX-FL)	Hydrochloric Acid 15–30% Sulfuric Acid 1–5% Non-Ionic Surfactants <1% Fragrance	S3
4	Caustic Soda Flakes	Sodium Hydroxide min. 98%	S4

## Data Availability

The raw data supporting the conclusions of this article will be made available by the authors on request.

## References

[1] Schneikert H. (1939). Das Verschwindenlassen der Leiche beim Mord. Arch. Kriminol..

[2] Cope D.J., Dupras T.L. (2009). The Effects of Household Corrosive Chemicals on Human Dentition. J. Forensic Sci..

[3] Schotsmans E.M.J., de Voorde W.V. (2017). Concealing the Crime: The Effects of Chemicals on Human Tissues. Taphonomy of Human Remains: Forensic Analysis of the Dead and the Depositional Environment.

[4] Udriştioiu L.A., Andrei M., Perde F., Curcă G.C. (2025). Postmortem tissue alterations induced by corrosive substances—A scoping review. J. Forensic Leg. Med..

[5] Ubelaker D.H., Haglund W.D.S., Sorg M.H. (2002). Taphonomic Applications in Forensic Anthropology. Forensic Taphonomy: The Postmortem Fate of Human Remains.

[6] D’Errico S., Turillazzi E., Pomara C., Fiore C., Monciotti F., Fineschi V. (2011). A Novel Macabre Ritual of the Italian Mafia (‘Ndrangheta): Covering Hands With Gloves and Burying the Corpse With Burnt Lime After Execution. Am. J. Forensic Med. Pathol..

[7] Vermeij E., Zoon P., van Wijk M., Gerretsen R. (2015). Microscopic Residues of Bone from Dissolving Human Remains in Acids. J. Forensic Sci..

[8] Blau S., Ranson D., Garvin H.M.L., Passalacqua N.V. (2019). Body in the Barrel: Complex Body Disposal and Recovery. Case Studies in Forensic Anthropology.

[9] Fulginiti L.C., Hartnett-McCann K.M., Di Modica F., Garvin H.M.L., Langley N.R. (2019). Sealed for Your Protection: A Triple Homicide Involving the Use of a Corrosive Agent to Obscure Identity. Case Studies in Forensic Anthropology.

[10] Hartnett K.M., Fulginiti L.C., Di Modica F. (2011). The Effects of Corrosive Substances on Human Bone, Teeth, Hair, Nails, and Soft Tissue. J. Forensic Sci..

[11] Gentile G., Tambuzzi S., Andreola S., Bailo P., Bilato G., Gorini I., Zoja R. (2021). Analysis of the corrosive effects of hydrochloric acid (HCl) on human bone: Preliminary microscopic study and observations for forensic purposes. Forensic Sci. Int..

[12] Lang J. (2002). Masking Identity: The Use of Corrosive and Caustic Agents on Bone and Dentition. Honors Thesis.

[13] Raj M., Boaz K., Srikant N. (2013). Are teeth evidence in acid environment. J. Forensic Dent. Sci..

[14] Seth R.K., Mishra G., Raj A., Sing S., Chaubey S. (2015). Effect of concentrated acids on soft and hard tissue: A macroscopic analysis. Univ. J. Dent. Sci..

[15] Damascena N.P., Santos-Filho M.V.C., de Souza G.R.B., da Silva L.A.F., Estevam C.S., Franco A., Paranhos L.R., Musse J.O. (2017). Testing the extraction of DNA from human teeth exposed to different chemical solutions. Int. J. Odontostomatol..

[16] Jones C.A., Bracewell T. (2022). Scanning electron microscopy (SEM) and macroscopic analysis of immature human permanent molar immersion in hydrochloric acid (HCL, 38%). J. Forensic Leg. Med..

[17] Trapp B.M., Tallman S.D. (2018). The effects of household corrosive substances on silver amalgam and porcelain-fused-to-metal restorations and non-restored teeth. Forensic Sci. Int..

[18] Ehrenberg R. (2011). Mafia Informants Fail Acid Test ScienceNews. https://www.sciencenews.org/article/mafia-informants-fail-acid-test.

[19] Jadhav K., Gupta N., Ahmed Mujib B., Amberkar V.S. (2009). Effect of acids on the teeth and its relevance in postmortem identification. J. Forensic Dent. Sci..

[20] Sowmya K., Sudheendra U., Khan S., Nagpal N., Prathamesh S. (2013). Assessment of morphological changes and DNA quantification: An: In vitro: Study on acid-immersed teeth. J. Forensic Dent. Sci..

[21] Jones C., Bracewell T., Torabi A., Beck C.C., Harvey T.B. (2020). The effect of hydrochloric acid (HCl) on permanent molars: A scanning electron microscope (SEM) and energy dispersive X-ray spectroscopy (EDS) study. Med. Sci. Law.

[22] Al-Owaidi M., Al-Terehi M.N., Al-Saadi A.H., Zibara K. (2020). Forensic STR identification of human teeth samples exposed to various acidic and alkaline chemical conditions in the Iraqi population. Syst. Rev. Pharm..

[23] Thurzo A., Jančovičová V., Hain M., Thurzo M., Novák B., Kosnáčová H., Lehotská V., Varga I., Kováč P., Moravanský N. (2022). Human Remains Identification Using Micro-CT, Chemometric and AI Methods in Forensic Experimental Reconstruction of Dental Patterns after Concentrated Sulphuric Acid Significant Impact. Molecules.

[24] Udriștioiu L.A., Dincă I., Curcă G.C. (2025). “Dissolving the Evidence”: A Forensic Experimental Study on Tissue Destruction and Trace Detection. Appl. Sci..

[25] Yadav P., Bishariya N., Lather J., Dhattarwal S.K., Sharma N., Lohhra A. (2025). Assessing the impact of corrosive acids on human bone integrity in forensic context. Forensic Sci. Med. Pathol..

[26] Udriştioiu L.A., Andrei M. (2026). Applicability of Dental Ground Sections in Forensic Science. Forensic Sci..

[27] Bracewell T., Jones C.A. (2025). Bridging methodological gaps in forensic science: A study of hydrochloric acid and human dentition. Forensic Sci. Int..

[28] Dorozhkin S.V. (2012). Dissolution mechanism of calcium apatites in acids: A review of literature. World J. Methodol..

[29] Guida A. (2006). Mechanism of action of sodium hypochlorite and its effects on dentin. Minerva Stomatol..

[30] Tartari T., Bachmann L., Maliza A.G., Andrade F.B., Duarte M.A., Bramante C.M. (2016). Tissue dissolution and modifications in dentin composition by different sodium hypochlorite concentrations. J. Appl. Oral Sci..

[31] Yamamoto T., Domon T., Takahashi S., Islam M.N., Suzuki R. (2000). The fibrous structure of the cemento-dentinal junction in human molars shown by scanning electron microscopy combined with NaOH-maceration. J. Periodontal Res..

[32] Monge J., Tillier A.-M., Mann A. (2006). Perikymata number and spacing on early modern human teeth: Evidence from Qafzeh cave, Israel. Bull. Mém. Soc. D’anthropol. Paris.

[33] Kumar G.S. (2023). Orban’s Oral Histology & Embryology.

[34] Aoba T., Yagi T. (1982). Effects of acid-dissolution on thin ground sections of enamel caries studied by microradiography and X-ray microbeam diffraction. J. Oral Pathol. Med..

[35] Marending M., Luder H.U., Brunner T.J., Knecht S., Stark W.J., Zehnder M. (2007). Effect of sodium hypochlorite on human root dentine—*Mechanical*, chemical and structural evaluation. Int. Endod. J..

[36] Wang T.F., Feng X.W., Gao Y.X., Wang M., Wang Y.N., Sa Y., Jiang T. (2017). Effects of different concentrations and exposure time of sodium hypochlorite on the structural, compositional and mechanical properties of human dentin. J. Huazhong Univ. Sci. Technol. [Med. Sci.].

[37] Gu L.S., Huang X.Q., Griffin B., Bergeron B.R., Pashley D.H., Niu L.N., Tay F.R. (2017). Primum non nocere—The effects of sodium hypochlorite on dentin as used in endodontics. Acta Biomater..

[38] Xu H., Ye Z., Zhang A., Lin F., Fu J., Fok A.S.L. (2022). Effects of concentration of sodium hypochlorite as an endodontic irrigant on the mechanical and structural properties of root dentine: A laboratory study. Int. Endod. J..

[39] Tonami K., Araki K., Mataki S., Kurosaki N. (2003). Effects of chloramines and sodium hypochlorite on carious dentin. J. Med. Dent. Sci..

[40] Feirstein S.R., Castagnola M.J., Bell D.M., Hassan M., Pujols A.M., Cabo L.L., Adserias-Garriga J., Zapico S.C. (2026). Assessing the Micro- and Macroscopic Changes of Chemically Altered Human Bone and Teeth. Biomolecules.

[41] Sarna-Boś K., Skic K., Boguta P., Adamczuk A., Vodanovic M., Chałas R. (2023). Elemental mapping of human teeth enamel, dentine and cementum in view of their microstructure. Micron.

[42] Kagayama M., Sasano Y., Mizoguchi I., Takahashi I. (1997). Confocal microscopy of cementocytes and their lacunae and canaliculi in rat molars. Anat. Embryol..

[43] Corte-Real A., Anjos M.J., Vieira D.M., Gamero J.J. (2012). The tooth for molecular analysis and identification: A forensic approach. J. Forensic Odontostomatol..

[44] Sluis I., Duijst W., Krap T. (2025). Bone histology for forensic anthropology: A technical review on the advances in microstructural analysis of taphonomically altered buried or subaerially exposed bone. Int. J. Leg. Med..

[45] Srinivasan M.R., Poorni S., Levin L., Dummer P.M., Nagendrababu V. (2025). New Numbering System for Teeth Following Hemisection, Bicuspidisation, and Root Resection. Int. Dent. J..

[46] Silva G.A., Moreira A., Alves J.B. (2011). Histological processing of teeth and periodontal tissues for light microscopy analysis. Methods Mol. Biol..

[47] Correia Sampaio F., Passos Farias I.A., Barros Mangueira Leite D.F., Avelino de Paiva M.A., Cavalcante da Costa A.D.P., Helvacıoğlu Kıvanç B. (2017). Dental Anatomical Features and Caries: A Relationship to be Investigated. Dental Anatomy.

[48] Janda R. (1995). Preparation of extracted natural human teeth for SEM investigations. Biomaterials.

